# Pressure pain threshold map of thoracolumbar paraspinal muscles after lengthening contractions in young male asymptomatic volunteers

**DOI:** 10.1038/s41598-022-20071-4

**Published:** 2022-09-22

**Authors:** Kohei Hanada, Hiroki Ota, Kazue Mizumura, Toru Taguchi

**Affiliations:** 1 Department of Physical Therapy, Faculty of Rehabilitation, Niigata University of Health and Welfare, 1398 Shimami-cho, Kita-ku, Niigata, 950-3198 Japan; 2 Institute for Human Movement and Medical Sciences (IHMMS), Niigata University of Health and Welfare, Niigata, 950-3198 Japan; 3 Department of Physiology, Nihon University School of Dentistry, Tokyo, 101-8310 Japan

**Keywords:** Neuroscience, Physiology, Biomarkers, Diseases, Health care, Health occupations, Medical research, Neurology, Signs and symptoms

## Abstract

This study aimed to characterise topographic distribution of pressure pain thresholds (PPTs) of thoracolumbar paraspinal muscles and its change after lengthening contractions (LCs) of the back muscles. Using young male asymptomatic participants in Experiment 1, we systematically examined the distribution of PPTs bilaterally in the range of Th1–L5 at measurement points 2 and 4 cm from the midline. PPTs were found to be higher in the lumbar segments of the paraspinal muscles than in the thoracic segments, and in muscles closer to the vertebrae (2 vs. 4 cm from the midline). The PPTs did not differ between the left and right sides in each segment. In Experiment 2, LC was applied by asking a part of participants recruited in Experiment 1 to fall their trunk from a starting position (parallel to the floor) to 40° flexed position, and then made it back as quickly as possible to the starting position. This cycle was repeated until participants could not keep contractions (30 times/set, 25.4 ± 10.6 sets). PPTs of the LC group decreased prominently in the lower thoracic and lumbar segments, and the decrease was more evident 24 h after LC compared to that 48 h after. In contrast, PPTs in the control group without LC remained unchanged. These results provided broad topographic images of PPTs in the thoracolumbar paraspinal muscles of young male participants with and without LC, and the obtained PPT maps could be a useful guide for better treatment of exercise-induced myofascial pain in the lower back.

## Introduction

Muscle pain is generally perceived in broad areas with a dull or acing sensation^1^, and chronic pain in the lower back muscles is highly prevalent in the general population^2,3^. As lower back muscle pain could restrict activities of daily living for many people including elderlies, labourers, and athletes, it is clinically important, especially in sports medicine and rehabilitation. According to an epidemiologic investigation in Japan, chronic musculoskeletal pain is more frequent in the lower back than in the extremities, such as elbow, hand, hip, knee, and foot^4^. From a histological point of view, the erector spinae muscle in the lower back contains more type I (slow) fibres than the vastus lateralis muscle (extremity muscle) does in humans^5,6^. Functionally, lower back muscles play a role in postural control rather than locomotion. Whether these differences in the fibre type compositions and functional roles are related with the high prevalence of chronic pain in lower back muscles, is unknown.

Delayed onset muscle soreness (DOMS), which is characterised by mechanical hyperalgesia/allodynia and commonly experienced after unaccustomed strenuous exercise including lengthening contraction (LC) of muscles^7–10^, has been proposed as a model for studying the mechanisms of clinical muscle pain, such as myofascial pain syndrome^11^. Myofascial pain syndrome is primarily characterised by trigger points (TrPs), which are defined as localised tender spots in a palpable taut band (hardened structure in the muscle). TrP-like sensitive spots in the hardened muscle/fascia tissues have been reported to be created after single and repetitive LC in rat, rabbit, and human models of DOMS^12–14^. Many researchers have investigated DOMS after a pioneering study by Hough^15^. However, most studies have focused on extremity muscles, such as the elbow flexors and knee extensors^16–18^, and few studies have targeted the paraspinal muscles in the lower back^19^.

Pressure pain threshold (PPT) can be a good measure of the magnitude of clinical muscle pain^20–23^, such as DOMS and low back pain with myofascial TrPs, since their most remarkable symptoms are mechanical hyperalgesia, which is perceived in response to compression, contraction, and stretch of affected muscles, while spontaneous pain (i.e., pain at rest) is rare in clinical muscle pain^11,24^. In a previous study, PPTs were measured at four lumbar sites (i.e. L2 and L4 segments bilaterally at approximately 2.5 cm from the spinous process), reporting increased pain sensitivity (DOMS) and reduced functional capacities of paraspinal muscles following flexion–extension exercises of the trunk^19^. However, the number of sites where PPTs were measured was limited; thus, it was not possible to build an entire topographic image of the PPT map in the thoracolumbar areas after LC of the back muscles. Binderup et al. reported that women had lower PPTs in the trapezius muscle of the cervicothoracic and lumbar areas compared to men^25^, and the topography in the trapezius muscle of the cervicothoracic area had considerable heterogeneity in the distribution under the DOMS condition, although the PPT topography has not been examined in the lumbar area^26^. Thus, using systematic PPT mapping approaches before and after LC in the present study, we tested the hypotheses that PPT topography of the paraspinal muscles in the thoracolumbar area had a certain heterogeneity both in the normal and mechanically-hyperalgesic conditions.

## Methods

### Participants

This study consisted of Experiments 1 and 2. In Experiment 1, a total of 40 young male asymptomatic volunteers was recruited (Table 1) to measure baseline PPTs without LC. Of these 40 participants, 24 were additionally involved in Experiment 2 (Table 2) to examine effects of LC on the PPTs. Actually, their baseline PPT data in Experiment 1 were taken from data before LC (i.e., 0 h after LC) in Experiment 2. The rest 16 participants of Experiment 1 were involved only in Experiment 1.

**Table 1:** Participant profiles in Experiment 1.

	Subjects (n = 40)	
	(Mean ± SD)	(Range)
Age (years)	20.9 ± 0.6	20–23
Height (cm)	172.9 ± 6.0	158–186
Body weight (kg)	65.8 ± 11.7	50–110
BMI	22.0 ± 3.3	18.0–34.3

**Table 2:** Participant profiles in Experiment 2.

	CTR (n = 12)		LC (n = 12)	
	(Mean ± SD)	(Range)	(Mean ± SD)	(Range)
Age (years)	20.9 ± 0.5	20–22	21.2 ± 0.8	20–23
Height (cm)	175.7 ± 5.9	168–186	171.5 ± 5.6	163–183
Body weight (kg)	67.3 ± 16.1	53–110	65.8 ± 9.0	50–80
BMI	21.7 ± 4.3	18.0–34.3	22.4 ± 2.8	18.4–27.4

Age, height, body weight, and body mass index (BMI) of the participants were matched so that they did not differ between the two groups (*p* > 0.05, Mann–Whitney U test).

All participants had no history of pain in the thoracolumbar area such that they consulted a doctor, and they were free from back pain on the day of the experiment. No participants who performed specific regular exercises on their back muscles were included in this study. None of the participants had therapeutic drugs or medications, which may have affected their perception of pain. The present study was approved by the Ethics Committee of Niigata University of Health and Welfare (Permission No. 18297-191115) and conducted in accordance with the Declaration of Helsinki. Written informed consent was obtained from all the participants prior to participation.

### Measurement of pressure pain threshold

**Figure 1:**
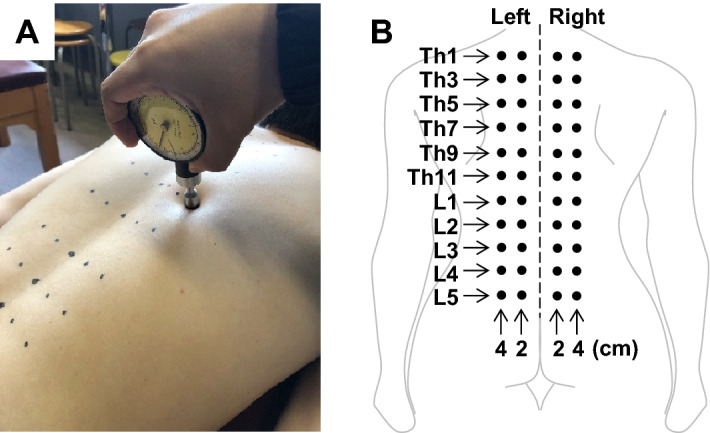
Measurement of pressure pain threshold in thoracolumbar paraspinal muscles. (**A**) Pressure algometry using a pressure algometer, having a flat and circular rubber tip with a surface of 1 cm^2^. (**B**) The measurement points. Bilateral paraspinal muscles of the thoracolumbar area (2 and 4 cm apart from midline at spinal segments Th1, Th3, Th5, Th7, Th9, Th11, L1, L2, L3, L4, and L5, i.e. 4 points × 11 segments = 44 points) were randomly tested to cancel order effect.

The participants lay on a bed in a prone position (Fig. 1A). Pressure pain threshold (PPT), which is defined as the minimum pressure inducing pain, was measured using a commercially available pressure algometer equipped with a flat and circular rubber tip of a surface of 1 cm^2^ (Pain Diagnostics and Thermography, NY, USA)^20,21,27^. Measurements were performed on the bilateral paraspinal muscles of the thoracolumbar area (2 and 4 cm from the midline at the spinal segments Th1, Th3, Th5, Th7, Th9, Th11, L1, L2, L3, L4, and L5; Fig. 1B). A total of 44 points (4 points × 11 segments) were marked with a pen and randomly tested once at one point^28,29^ via random number generation, to minimise the order effect of the measurements. Pressure stimuli were applied as perpendicularly as possible to the muscle via the skin surface at a speed of approximately 98 kPa/sec^30^, until participants perceived pain from the stimuli. The perpendicular stimuli enabled to minimise the edge effect of a flat and circular rubber tip of the algometer. Measurements were performed approximately every 20 s to avoid temporal summation^31^. It took approximately 15 min for the measurement of one participant on one experimental day. The maximum value, which was defined by the algometer used in this study, was 980 kPa, but the PPTs in all measurements were within the range of 980 kPa. In Experiment 1, pressure algometry was performed in asymptomatic participants without any intervention.

### Lengthening contractions of back muscles

In Experiment 2, 24 young male asymptomatic participants were recruited to further examine the effects of lengthening contraction (LC) on the PPT map of the thoracolumbar area. Their baseline PPT data before LC were included in Experiment 1 as described above (see ‘Participants’ section). The 24 participants were assigned to either the control (CTR, n = 12) or LC group (n = 12). Participants were matched by age, height, body weight, body mass index (BMI), and baseline PPT values before LC, to ensure homogeneity between the two groups (Table 2 and Supplementary Table 2).

**Figure 2:**
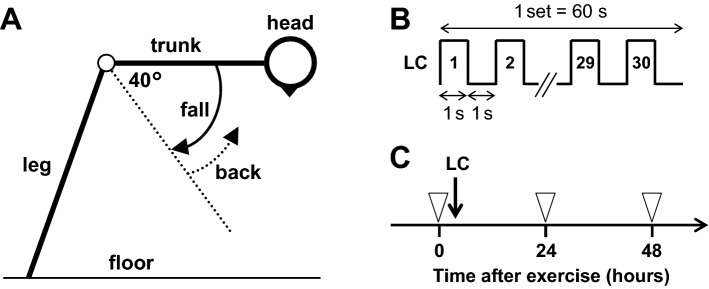
Lengthening contraction (LC). (**A**) Schematic illustration of LC. Using a back extension bench, participants allowed their trunk to fall from a starting position (parallel to the floor) to 40° flexion position, and then returned to the starting position. This “fall and back” cycle was performed in 1 s, and followed by a rest period of 1 s. During the rest period, an experimenter held the subject’s trunk at the starting position. (**B**) Protocol for LC. Using a sound-guided metronome, the LC cycle was repeated 30 times in one set (i.e. 2 s × 30 times = 60 s). The LC set was repeated with inter-set intervals of 1 min until participants could not continue the exercise. (**C**) Experimental schedule. Pressure pain threshold was measured 0 (before), 24, and 48 h after LC.

Using a back extension bench (Trust, Fighting Road Co., Ltd.), participants allowed their trunk to fall from a starting position (parallel to the floor) to 40° flexion position, and then made it back as quickly as possible to the starting position (Fig. 2A). This cycle was performed for 1 s, followed by a rest period of 1 s (Fig. 2B). During the rest period, an experimenter held the participants’ trunk at the starting position to allow the participants to rest. Using a sound-guided metronome, the LC cycle was repeated 30 times in one set (60 s). The LC set was repeated with inter-set intervals of 1 min until the participants could not continue the exercise. To examine the effects of LC on PPTs, measurements were repeated 0 (before), 24, and 48 h after LC (Fig. 2C). Young male asymptomatic participants without LC loading served as CTR.

To examine chronological changes of DOMS, we normalised raw PPT values obtained 24, and 48 h after LC from individual participants as % changes of PPTs based on the data before LC (baseline) (see detailed data in Fig. 6). The normalisation was needed since raw PPTs varied considerably across participants and measurement sites (i.e. vertebral segments or distance from midline). Based on the normalized chronological changes of PPTs in Fig. 6, we quantified the magnitude of DOMS from the area under the curve (AUC) at all 44 points tested (see detailed data in Fig. 7). The AUC was calculated for individual participants as follows:

$${\text{AUC}}\;(\% ) = \left({\%} \;{\text{changes}}\;{\text{of}}\;{\text{PPTs}}\;{\text{at}}\;24\;{\text{h}}\;{\text{from}}\;{\text{the}}\;{\text{baseline}}\;{\text{before}}\;{\text{LC}} + {\text{those}}\;{\text{at}}\;48\;{\text{h}} \right)/ 2$$

### Statistical analyses

Data are expressed as mean ± SD. Normality of all data sets was checked using the Shapiro–Wilk test before choosing parametric or non-parametric analysis. In Experiment 1, PPT values 2 and 4 cm from the midline on the left and right side were compared using the Friedman test followed by Dunn’s multiple comparison test. In Experiment 2, participant profiles between the CTR and LC groups were compared using the Mann–Whitney U-test. Comparisons of normalised PPTs with time between the two groups were performed using two-way repeated measures analysis of variance (ANOVA) followed by Sidak’s multiple comparison test. *p* < 0.05 was considered statistically significant.

## Results

### PPTs in thoracolumbar paraspinal muscles of participants without LC

**Figure 3:**
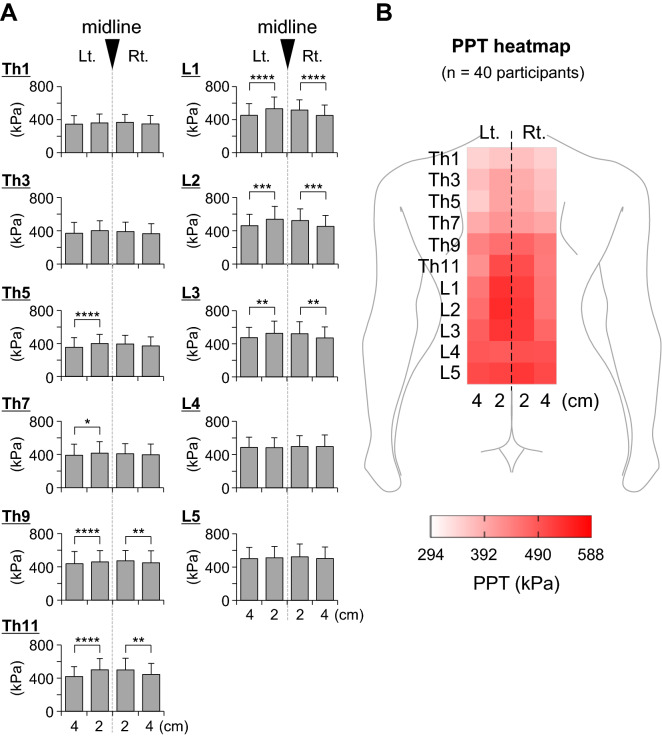
Pressure pain threshold (PPT) of paraspinal muscles in participants without LC. **A.** PPT measured in the thoracolumbar area (2 and 4 cm apart from midline vertebrae at the spinal segments Th1, Th3, Th5, Th7, Th9, Th11, L1, L2, L3, L4, and L5; n = 40 participants). See Supplementary Table 1 for detailed PPT values. Note tendencies for higher PPTs in lumbar compared with thoracic segments, and in muscles 2 cm apart from the vertebrae compared to those 4 cm at segments Th5–L3 (*p < 0.05, **p < 0.01, ***p < 0.001, and ****p < 0.0001, Friedman test followed by Dunn’s multiple comparison test). No significant differences in PPTs were observed between the left and the right side in any spinal segments examined (left 2 cm vs. right 2 cm and left 4 cm vs. right 4 cm, p > 0.05, Friedman test followed by Dunn’s multiple comparison test). **B.** A heatmap image of the mean PPTs obtained in the measurement in **A**. Mean PPTs ranged from 294 to 588 kPa.

In Experiment 1, PPTs in young male asymptomatic participants without LC varied depending on the participants and measurement sites (thoracic vs. lumbar segment or distance from midline). Overall, the mean PPTs of the paraspinal muscles in the thoracolumbar area ranged from 294 to 588 kPa (Fig. 3A,B, see Supplementary Table 1 for detailed PPT values**)**. In general, the following were observed: (1) PPTs tended to be higher in the lumbar segments compared to the thoracic segments, and showed a gradual increase toward the caudal direction in the thoracic area, (2) PPTs were higher in the paraspinal muscles 2 cm apart from the vertebrae compared with 4 cm, especially at segments Th5–L3 (left 2 vs. 4 cm and right 2 vs. 4 cm, p < 0.05 ~ 0.0001, Friedman test followed by Dunn’s multiple comparison test, Fig. 3A), and (3) no significant left–right differences in PPTs were observed in any of spinal segments examined (left 2 cm vs. right 2 cm and left 4 cm vs. right 4 cm, p > 0.05, Friedman test followed by Dunn’s multiple comparison test, Fig. 3A).

### Changes in PPTs after lengthening contractions

**Figure 4:**
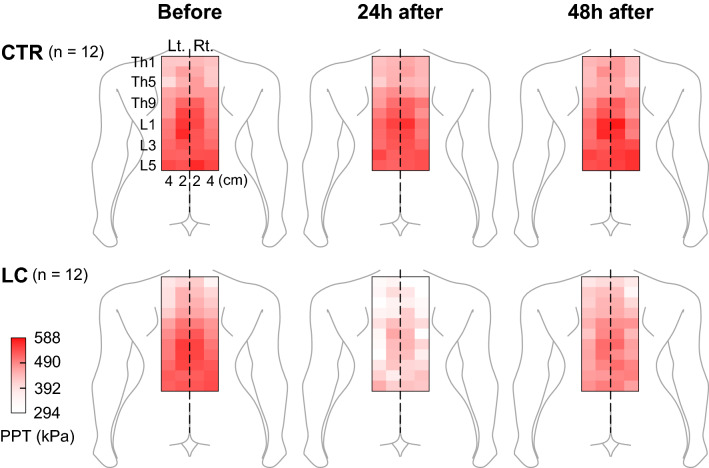
Heatmap images of pressure pain thresholds (PPTs) after lengthening contractions (LCs). Note a remarkable decrease in PPTs 24 h after LC in the LC group, which appeared to recover 48 h after LC. In the CTR group, PPT maps appeared to be the same 0 (before), 24, and 48 h after LC. Mean PPTs ranged from 294 to 588 kPa. See Supplementary Table 2 for detailed PPT values.

In Experiment 2 of the present study, we examined the effects of LC on the PPTs of paraspinal muscles in the thoracolumbar area of young male asymptomatic participants. Participants in the LC group (n = 12) performed repetitive LCs of the back muscles until they could not continue the task. The number of LC sets was 25.4 ± 10.6 (range: 11–42 sets). Figure 4 illustrates a heatmap of PPTs 0 (before), 24, and 48 h after LC. Although PPTs in the CTR group remained unchanged over time, decreases in PPTs were observed 24 h after LC. The decreased PPTs recovered 48 h after LC in many points with some points remained decreased (see Supplementary Table 2 for detailed mean PPT values).

**Figure 5:**
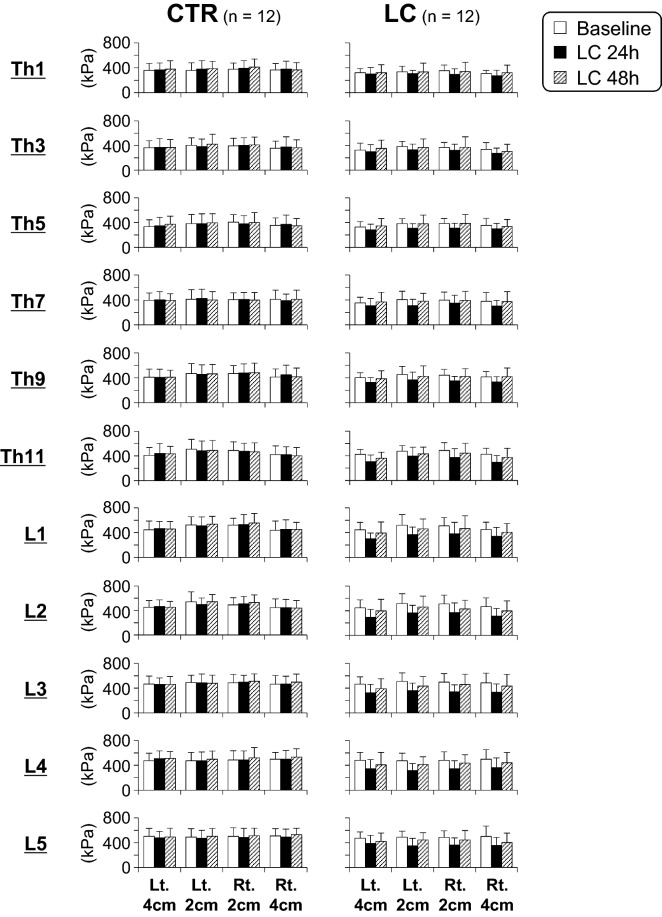
Changes in pressure pain thresholds (PPTs) after lengthening contractions (LCs). PPTs in the thoracolumbar paraspinal muscles 0 (before), 24, and 48 h after LCs. Data are shown as mean ± SD in the CTR (n = 12) and LC groups (n = 12). See Supplementary Table 2 for detailed PPT values. Note that PPTs decreased 24 h after LC in the LC group, and that the decrease was prominent at segments from the lower thoracic to lumbar areas. In contrast, PPTs in the CTR group remained unchanged over time.

In Fig. 5, changes in the raw PPTs at 44 measurement sites (4 × 11 segments) are shown in the CTR and LC groups. The PPTs tended to decrease 24 h after LC in the LC group, and the decrease appeared to be prominent at lower thoracic and lumbar segments compared to the upper and middle thoracic segments (see Supplementary Table 2 for detailed PPT values).

**Figure 6:**
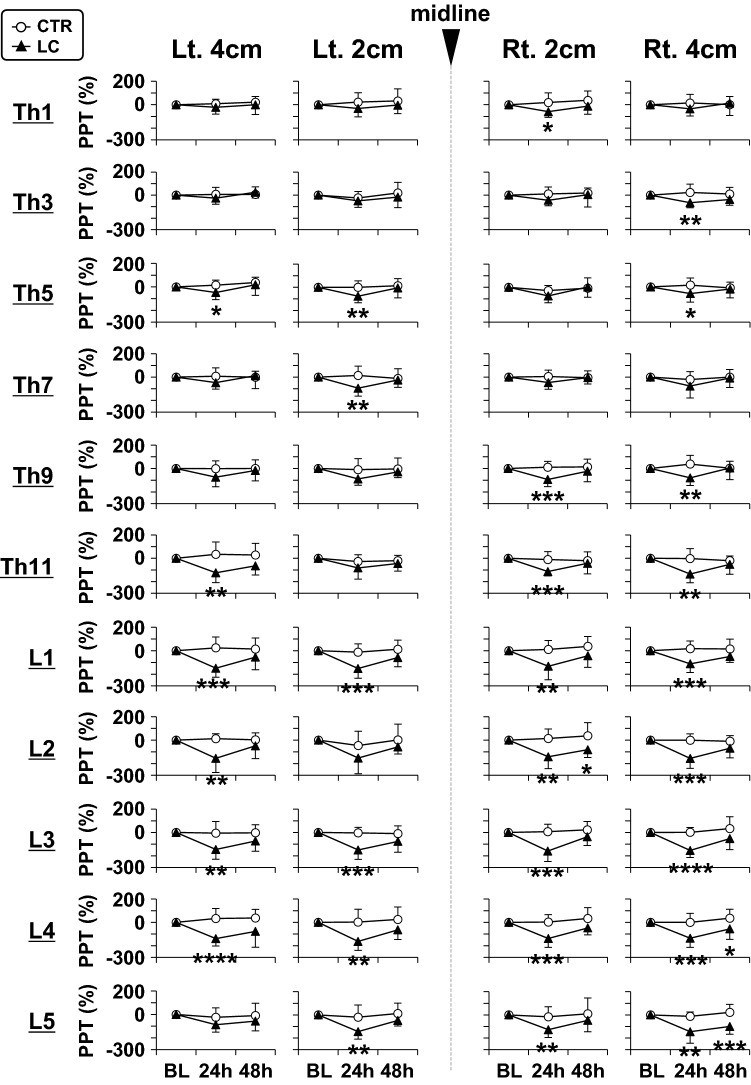
Normalized changes in pressure pain thresholds (PPTs) after lengthening contractions (LCs). PPTs were normalised in the CTR (n = 12) and LC (n = 12) groups. The mean PPTs in each group before LC were set to 0%. *p < 0.05, **p < 0.01, ***p < 0.001, and ****p < 0.0001, CTR vs. LC at each time point of measurement, two-way repeated measures ANOVA followed by Sidak’s multiple comparison test.

Since the baseline PPTs before LC and changes in PPTs after LC varied considerably across measurement sites (i.e. vertebral segments or distance from midline) as shown in Fig. 5 and Supplementary Table 2, raw PPTs measured after LC were normalised based on the data before LC (Fig. 6). By normalisation, decreases in PPTs were more obvious in lumbar segments than in thoracic segments (p < 0.05 ~ 0.0001, CTR vs. LC, two-way repeated measures ANOVA with Geisser–Greenhouse correction followed by Sidak’s multiple comparison test). In most cases, the decrease in normalised PPTs appeared to recover 48 h after LC.

**Figure 7:**
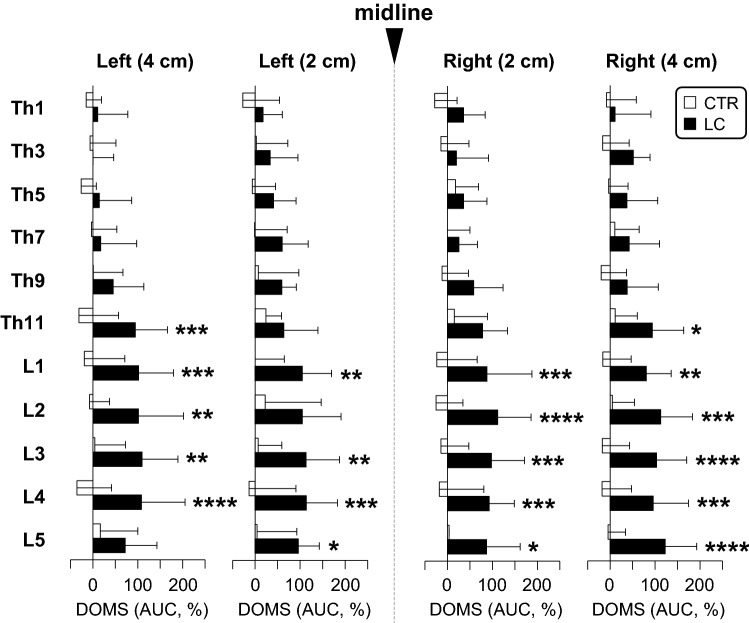
Magnitude of DOMS in the thoracolumbar areas after lengthening contractions (LCs). The area under the curve (AUC) of normalised PPTs, which was calculated from data at 0 (BL), 24, and 48 h in Fig. 6, was used as the magnitude of the DOMS in each vertebral segment of four measurement points (i.e. left 4 cm, left 2 cm, right 2 cm, and right 4 cm). Note significantly higher magnitude of DOMS in the LC group than in the CTR at segments Th11–L5 (*p < 0.05, **p < 0.01, ***p < 0.001, and ****p < 0.0001, CTR (n = 12) vs. LC (n = 12), two-way repeated measures ANOVA followed by Sidak’s multiple comparison test).

Figure 7 shows magnitude of DOMS in the thoracolumbar areas induced after LC (see “Methods” for calculation of the magnitude of DOMS using the AUC of normalized PPTs shown in Fig. 6). The AUC was significantly greater in the LC group than in the CTR, at lower thoracic and lumbar segments of Th11–L5, peaking at around L3 (*p* < 0.05 ~ 0.0001, two-way repeated measures ANOVA followed by Sidak’s multiple comparison test, see Supplementary Table 3 for statistical summary). No obvious mediolateral preference in changes of the AUC was observed at the lower thoracic and lumbar segments.

Using the G*Power software (ver. 3.1.9.7), we calculated the achieved power (1–β err prob) based on the sample size used in the present study, where “effect size f” and “α err prob” were set at 0.4 and 0.05, respectively. The power value (1–β err prob) was 0.631.

## Discussion

Using systematic pressure algometry with young male asymptomatic participants in the present study, we showed the distribution of pressure pain thresholds in the thoracolumbar paraspinal muscles. Heatmap images of PPTs in participants who underwent LC of the back-extension muscles were also produced. We found that the PPT topographic map had a certain heterogeneity in the normal condition with asymptomatic participants, and the topographic map exhibited visible changes in muscles, especially at lower thoracic and lumbar segments of the mechanically-hyperalgesic condition (DOMS). These mapping approaches provided a useful guide for better treatment of exercise-induced myofascial pain in the lower back.

### Topographic mapping of PPTs

PPT has been widely used as a valid measure to quantify the magnitude of clinical and experimental muscle pain such as that in DOMS and myofascial pain with trigger points^20–23,27^, since the most characteristic symptom of muscle pain is mechanical hyperalgesia^11,24^. In the present study, we used a commercially available pressure algometer with a flat and circular rubber tip of a surface of 1 cm^2^ to measure muscle PPT according to previous studies^20,21,23,24,27^. A pressure algometer equipped with a large-sized probe is assumed to be appropriate for measuring the pain threshold in deep tissues, such as muscles, both in humans^32^ and rodents^33,34^. Finocchietti et al. reported that PPT is mainly associated with strain in the muscle, and that pressure-induced muscle pain is most efficiently induced by large and rounded (hemispherical) probes, while smaller and flat ones preferentially activate superficial structures^35^. Thus, in this experiment, we cannot exclude some inclusion of superficial components from the measured PPTs using a flat tip. This point needs to be noted as a study limitation. However, the tip effect could have been minimised since it is made of rubber, and since the tip was applied as perpendicularly as possible to the muscle via the skin surface, so that the edge effect could be minimised.

To create topographic mapping of PPTs, grids for measurement are defined as reported previously in the temporalis muscle^36^, trapezius muscle^26^, infraspinatus muscle^37^, shoulder^38^, hand^39^, lower extremity^40^, knee^41^, and foot^42^. Alburquerque-Sendin et al. suggested the importance of the following for measurement using the grids: (1) number of measurement sites of PPT, (2) measuring order, (3) interstimulus time interval, and 4) duration of the measurement^40^. Consideration of these points can minimise the spatial and temporal summation of pain induced by repeated mechanical stimulations close to one another, the order effect of the measurement, and boredom or a lack of alertness^31,40,43^.

In the present study, we made grids consisting of 44 measurement sites (4 × 11 spinal segments) in the paraspinal muscles of the thoracolumbar area. Using young male asymptomatic participants without LC in Experiment 1, the PPTs of the paraspinal muscles at the 44 measurement sites were randomly tested. The time interval between the measurement trials was approximately 20 s and the total duration of the measurement for one participant per experiment was approximately 15 min. Thus, these procedures and variables used in this study were within the range reported in previous studies^31,40^.

### PPT maps in asymptomatic participants without LC

In Experiment 1 of the present study, the thoracolumbar paraspinal muscles of young male asymptomatic participants without LC exhibited PPTs ranging between 294 and 588 kPa; these were similar to those reported by Binderup et al.^25^, but relatively low compared to those reported by Farasyn and Meeusen^30^. Since these studies used the same algometer, the difference in the PPT values might have resulted from variation in the measurement sites (e.g. distance from vertebrae) or characteristics of participants, such as age or BMI. In the present study, we found the following observations in participants without LC: 1) PPTs were higher in lumbar segments than in thoracic segments, 2) PPTs were higher in paraspinal muscles close to vertebrae (2 cm from the midline) than in those far from vertebrae (4 cm from the midline), especially at segments Th5–L3, and 3) PPTs did not differ between the left and right sides in any thoracolumbar segments examined.

The higher PPTs in the lumbar segments than in the thoracic segments were consistent with previous studies^25,30^. A gradual increase in PPTs toward the caudal direction along the spine in the thoracic area was also in line with a previous study^44^. Anatomically, the trapezius muscle, which composes the shallowest layer under the back skin, is distributed over the broad cervicothoracic area, while the thoracolumbar fascia (TLF) and aponeurosis of the latissimus dorsi muscle, which both contain massive collagen fibres, cover the superficial lumbar area^45^. Since musculotendinous or collagenous sites were less sensitive to pressure stimulus than the muscle belly sites^46,47^, this explains the exhibition of higher PPTs in the lumbar area during the present study. The distribution and sensitivity of nociceptive afferents in the tissues of the thoracolumbar area can also be used as the neurophysiological basis for determining PPTs. The lumbar region of the TLF is densely innervated by nociceptive afferent fibres in humans and rodents^48–51^. In rats, spinal dorsal horn neurones receive input from the TLF^52,53^. In human participants, intrafascial injection of hypertonic saline into the TLF induced a greater and longer pain sensation compared with injection into the subcutaneous tissue or muscle^54^. These findings suggest that the fascia in the thoracolumbar area is an important source of pain perception, which potentially affects PPTs. However, no data are available to compare the distribution and sensitivity of nociceptive afferents between the thoracic and lumbar areas of the TLF in humans.

As for the second observation, higher PPTs in the paraspinal muscles located close to the vertebrae were in line with previous studies^25,26,55^. In animal experiments, high-threshold nociceptive afferents terminate primarily in the periosteum and synovium, and normally respond only to noxious stimuli^56^, whereas mechanosensitive afferents innervating the paravertebral muscles have a relatively low mechanical response threshold^57^. These differences in the mechanical response thresholds of nociceptors might be associated with higher PPTs in the paraspinal muscles located close to the vertebrae, as observed in this study.

As to the third observation, similar PPT values between the left and the right side in paraspinal muscles of the thoracolumbar area were also reported in other muscles, such as the trapezius muscle and low back muscles^19,25,55^. In the present study, we randomly measured PPTs at all 44 sites. Randomisation could minimise spatial and temporal summation and order effects to cancel left–right differences of PPTs.

### PPT maps in participants exposed to LC

In the current study, young male asymptomatic participants were subjected to repetitive LC to examine changes in topographic PPT maps in DOMS. To induce DOMS in the back trunk muscles, no standard protocol is available at present, that is, researchers have used variable LC protocols with different loads, speed of contractions/stretches, number of repetitions, inter-set intervals, etc.^19,58,59^. We used faster LC in the present study, where the back muscles were contracted as quickly as possible against participants’ weight to move their trunk back to the starting position, because the faster stretch speed of LC was the critical determinant for increased DOMS intensity not only in humans^60^, but also in rats^61^.

Using topographic mapping approaches, we found that the LC decreased the overall PPTs in the paraspinal muscle of the thoracolumbar area. The decreased PPTs were prominent 24 h after LC, and tended to recover 48 h post-exercise, as in previous studies^19,59^. Since absolute PPT values varied among different spinal segments and distance from the vertebrae as described above, we normalised the PPTs to examine relative changes in the magnitude of DOMS. The analysis revealed that DOMS occurred even in the lower thoracic and all lumbar segments of the paraspinal muscles, and the rostrocaudal distribution was found to be much wider than that reported in a previous study^19^, where PPTs have been measured only at the lumbar segments L2 and L4 bilaterally. No mediolateral preferences in the distribution of DOMS could be seen in any spinal segment examined. Schellenberg and colleagues have reported that range of motion was wider and electromyographic activity was greater in the lower back than in the upper back during trunk extension exercises^62^. These results indicate that mechanical loading during the course of LC was much stronger at the lower thoracic and lumbar segments to induce the greater mechanical hyperalgesia compared to the upper and middle thoracic segments without mediolateral preferences at each segment, since the intensity of mechanical loading have been reported to determine the magnitude of DOMS^61^. The topographic distribution of mechanical hyperalgesia revealed by the present study could be clinically important for therapeutic strategies of myofascial pain in the lower back after exercise (DOMS).

### Limitations

Since we have recruited only young male asymptomatic participants to obtain topographic mappings of PPTs in the thoracolumbar paraspinal muscles, we cannot tell whether the results observed in the present study were extrapolated for female or elderly participants or patients with low back pain.

From a statistical point of view, the achieved power (1–β err prob) calculated from the actual sample size (n = 12 participants each in the CTR and the LC group) was slightly small in Experiment 2, although effects of LC on the topographic distribution of PPTs could be detected as shown in the present study.

## Conclusion

Using systematic PPT mapping approaches with young male asymptomatic participants in the present study, we found that PPT topography of the thoracolumbar paraspinal muscles had a certain heterogeneity both in normal and hyperalgesic conditions with and without LC, and that the LC-induced mechanical hyperalgesia occurred in the lower thoracic and all lumbar segments of the paraspinal muscles. These observations could be a useful guide for better understanding and treatment of muscle pain after exercise (DOMS) in the lower back.

## Supplementary Information

Supplementary Table 1.

Supplementary Table 2.

Supplementary Table 3.

## Data Availability

Requests for materials should be addressed to T.T.
