# Supplementary material for: Pressure pain threshold map of thoracolumbar paraspinal muscles after lengthening contractions in young male asymptomatic volunteers
Source: Sci Rep. 2022 Sep 22;12:15825. doi: 10.1038/s41598-022-20071-4 (PMC9499944; doi:10.1038/s41598-022-20071-4)
Supplement: Supplementary file 1 — Supplementary Table 1. [file 41598_2022_20071_MOESM1_ESM.pptx]

## Slide 1
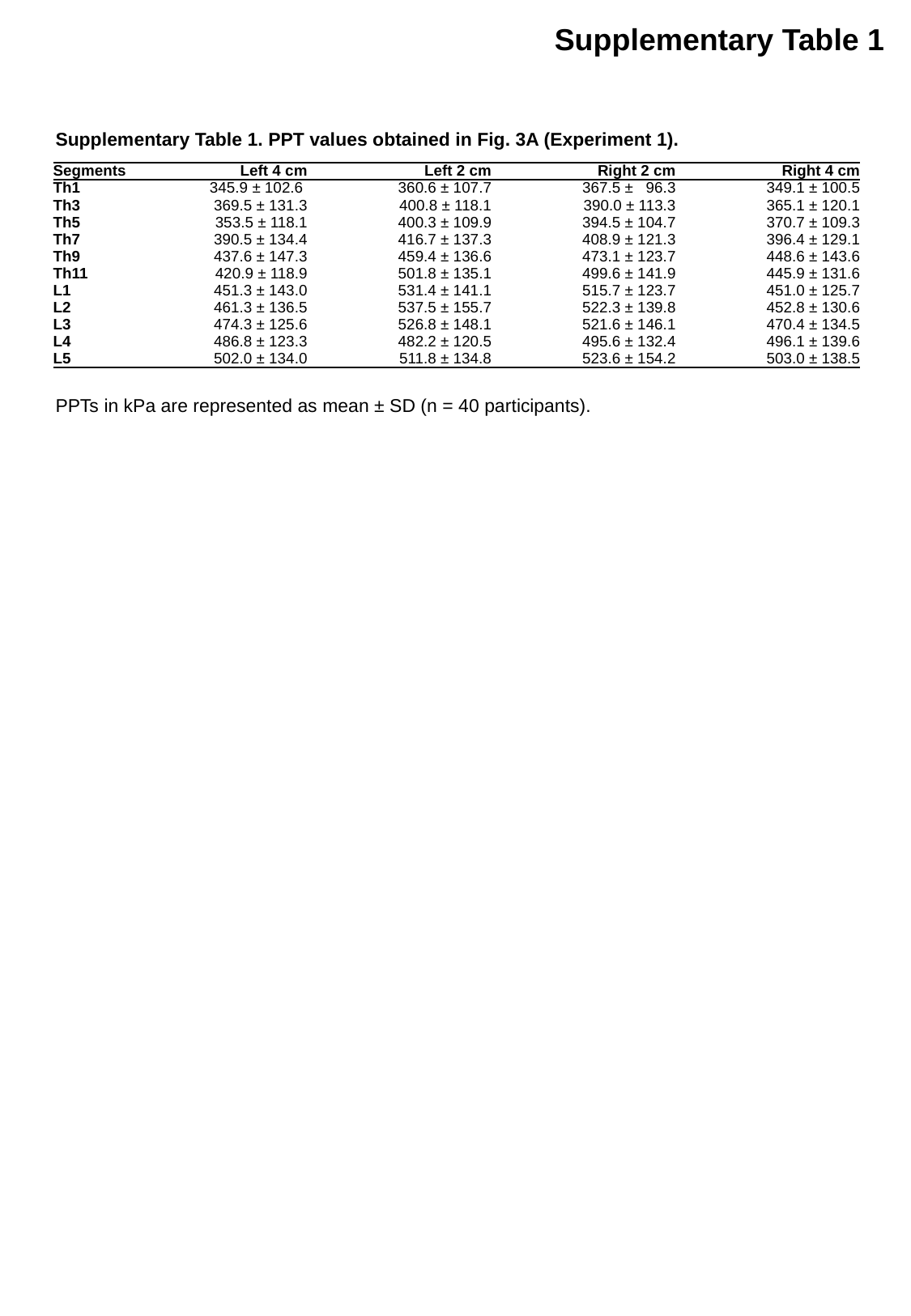

Supplementary Table 1
Supplementary Table 1. PPT values obtained in Fig. 3A (Experiment 1).
| Segments | Left 4 cm | Left 2 cm | Right 2 cm | Right 4 cm |
| --- | --- | --- | --- | --- |
| Th1 | 345.9 ± 102.6 | 360.6 ± 107.7 | 367.5 ± 96.3 | 349.1 ± 100.5 |
| Th3 | 369.5 ± 131.3 | 400.8 ± 118.1 | 390.0 ± 113.3 | 365.1 ± 120.1 |
| Th5 | 353.5 ± 118.1 | 400.3 ± 109.9 | 394.5 ± 104.7 | 370.7 ± 109.3 |
| Th7 | 390.5 ± 134.4 | 416.7 ± 137.3 | 408.9 ± 121.3 | 396.4 ± 129.1 |
| Th9 | 437.6 ± 147.3 | 459.4 ± 136.6 | 473.1 ± 123.7 | 448.6 ± 143.6 |
| Th11 | 420.9 ± 118.9 | 501.8 ± 135.1 | 499.6 ± 141.9 | 445.9 ± 131.6 |
| L1 | 451.3 ± 143.0 | 531.4 ± 141.1 | 515.7 ± 123.7 | 451.0 ± 125.7 |
| L2 | 461.3 ± 136.5 | 537.5 ± 155.7 | 522.3 ± 139.8 | 452.8 ± 130.6 |
| L3 | 474.3 ± 125.6 | 526.8 ± 148.1 | 521.6 ± 146.1 | 470.4 ± 134.5 |
| L4 | 486.8 ± 123.3 | 482.2 ± 120.5 | 495.6 ± 132.4 | 496.1 ± 139.6 |
| L5 | 502.0 ± 134.0 | 511.8 ± 134.8 | 523.6 ± 154.2 | 503.0 ± 138.5 |
PPTs in kPa are represented as mean ± SD (n = 40 participants).
