# Supplementary material for: Pressure pain threshold map of thoracolumbar paraspinal muscles after lengthening contractions in young male asymptomatic volunteers
Source: Sci Rep. 2022 Sep 22;12:15825. doi: 10.1038/s41598-022-20071-4 (PMC9499944; doi:10.1038/s41598-022-20071-4)
Supplement: Supplementary file 2 — Supplementary Table 2. [file 41598_2022_20071_MOESM2_ESM.pptx]

## Slide 1
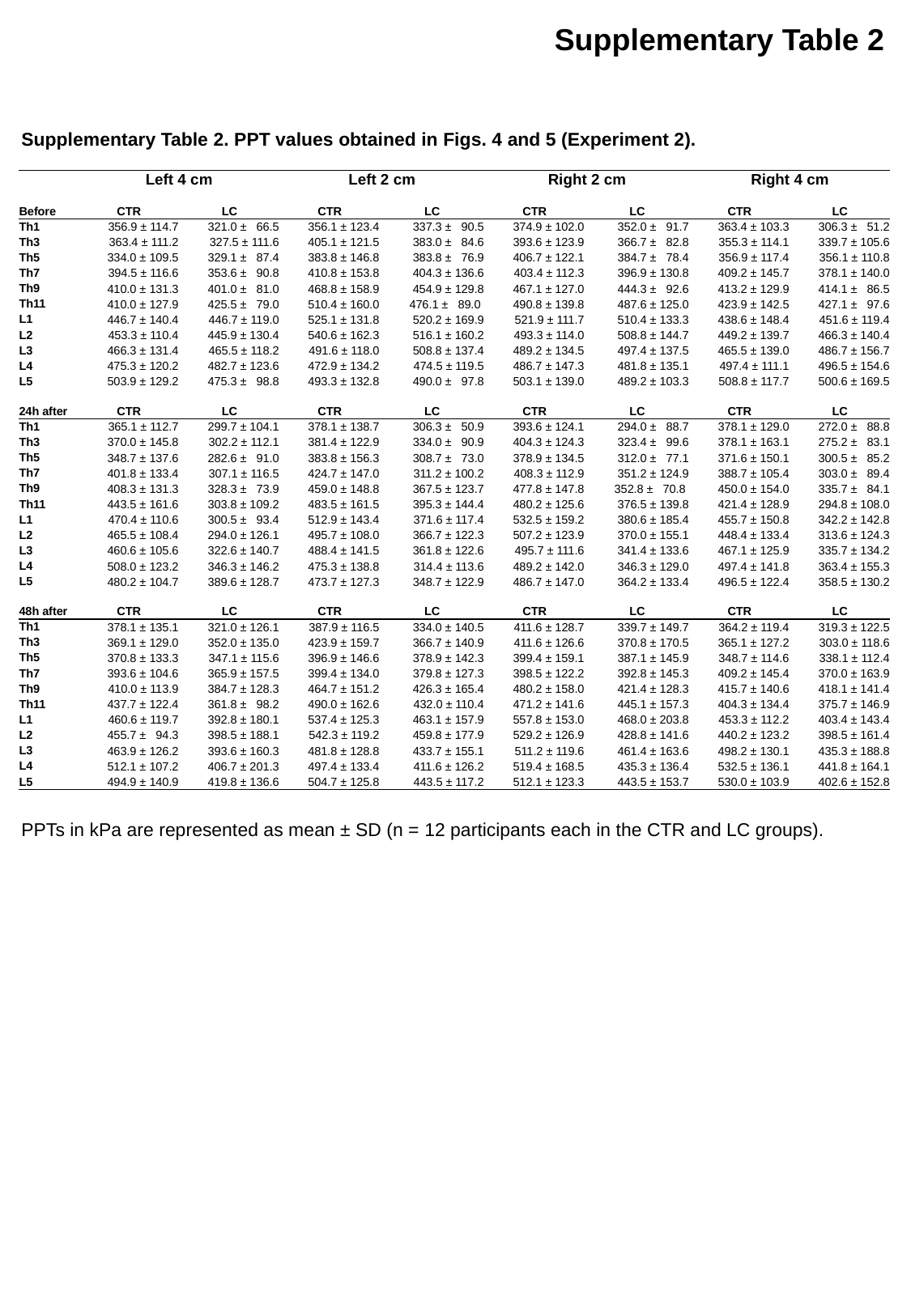

Supplementary Table 2
Supplementary Table 2. PPT values obtained in Figs. 4 and 5 (Experiment 2).
| | Left 4 cm | | Left 2 cm | | Right 2 cm | | Right 4 cm | |
| --- | --- | --- | --- | --- | --- | --- | --- | --- |
| | | | | | | | | |
| Before | CTR | LC | CTR | LC | CTR | LC | CTR | LC |
| Th1 | 356.9 ± 114.7 | 321.0 ± 66.5 | 356.1 ± 123.4 | 337.3 ± 90.5 | 374.9 ± 102.0 | 352.0 ± 91.7 | 363.4 ± 103.3 | 306.3 ± 51.2 |
| Th3 | 363.4 ± 111.2 | 327.5 ± 111.6 | 405.1 ± 121.5 | 383.0 ± 84.6 | 393.6 ± 123.9 | 366.7 ± 82.8 | 355.3 ± 114.1 | 339.7 ± 105.6 |
| Th5 | 334.0 ± 109.5 | 329.1 ± 87.4 | 383.8 ± 146.8 | 383.8 ± 76.9 | 406.7 ± 122.1 | 384.7 ± 78.4 | 356.9 ± 117.4 | 356.1 ± 110.8 |
| Th7 | 394.5 ± 116.6 | 353.6 ± 90.8 | 410.8 ± 153.8 | 404.3 ± 136.6 | 403.4 ± 112.3 | 396.9 ± 130.8 | 409.2 ± 145.7 | 378.1 ± 140.0 |
| Th9 | 410.0 ± 131.3 | 401.0 ± 81.0 | 468.8 ± 158.9 | 454.9 ± 129.8 | 467.1 ± 127.0 | 444.3 ± 92.6 | 413.2 ± 129.9 | 414.1 ± 86.5 |
| Th11 | 410.0 ± 127.9 | 425.5 ± 79.0 | 510.4 ± 160.0 | 476.1 ± 89.0 | 490.8 ± 139.8 | 487.6 ± 125.0 | 423.9 ± 142.5 | 427.1 ± 97.6 |
| L1 | 446.7 ± 140.4 | 446.7 ± 119.0 | 525.1 ± 131.8 | 520.2 ± 169.9 | 521.9 ± 111.7 | 510.4 ± 133.3 | 438.6 ± 148.4 | 451.6 ± 119.4 |
| L2 | 453.3 ± 110.4 | 445.9 ± 130.4 | 540.6 ± 162.3 | 516.1 ± 160.2 | 493.3 ± 114.0 | 508.8 ± 144.7 | 449.2 ± 139.7 | 466.3 ± 140.4 |
| L3 | 466.3 ± 131.4 | 465.5 ± 118.2 | 491.6 ± 118.0 | 508.8 ± 137.4 | 489.2 ± 134.5 | 497.4 ± 137.5 | 465.5 ± 139.0 | 486.7 ± 156.7 |
| L4 | 475.3 ± 120.2 | 482.7 ± 123.6 | 472.9 ± 134.2 | 474.5 ± 119.5 | 486.7 ± 147.3 | 481.8 ± 135.1 | 497.4 ± 111.1 | 496.5 ± 154.6 |
| L5 | 503.9 ± 129.2 | 475.3 ± 98.8 | 493.3 ± 132.8 | 490.0 ± 97.8 | 503.1 ± 139.0 | 489.2 ± 103.3 | 508.8 ± 117.7 | 500.6 ± 169.5 |
| | | | | | | | | |
| 24h after | CTR | LC | CTR | LC | CTR | LC | CTR | LC |
| Th1 | 365.1 ± 112.7 | 299.7 ± 104.1 | 378.1 ± 138.7 | 306.3 ± 50.9 | 393.6 ± 124.1 | 294.0 ± 88.7 | 378.1 ± 129.0 | 272.0 ± 88.8 |
| Th3 | 370.0 ± 145.8 | 302.2 ± 112.1 | 381.4 ± 122.9 | 334.0 ± 90.9 | 404.3 ± 124.3 | 323.4 ± 99.6 | 378.1 ± 163.1 | 275.2 ± 83.1 |
| Th5 | 348.7 ± 137.6 | 282.6 ± 91.0 | 383.8 ± 156.3 | 308.7 ± 73.0 | 378.9 ± 134.5 | 312.0 ± 77.1 | 371.6 ± 150.1 | 300.5 ± 85.2 |
| Th7 | 401.8 ± 133.4 | 307.1 ± 116.5 | 424.7 ± 147.0 | 311.2 ± 100.2 | 408.3 ± 112.9 | 351.2 ± 124.9 | 388.7 ± 105.4 | 303.0 ± 89.4 |
| Th9 | 408.3 ± 131.3 | 328.3 ± 73.9 | 459.0 ± 148.8 | 367.5 ± 123.7 | 477.8 ± 147.8 | 352.8 ± 70.8 | 450.0 ± 154.0 | 335.7 ± 84.1 |
| Th11 | 443.5 ± 161.6 | 303.8 ± 109.2 | 483.5 ± 161.5 | 395.3 ± 144.4 | 480.2 ± 125.6 | 376.5 ± 139.8 | 421.4 ± 128.9 | 294.8 ± 108.0 |
| L1 | 470.4 ± 110.6 | 300.5 ± 93.4 | 512.9 ± 143.4 | 371.6 ± 117.4 | 532.5 ± 159.2 | 380.6 ± 185.4 | 455.7 ± 150.8 | 342.2 ± 142.8 |
| L2 | 465.5 ± 108.4 | 294.0 ± 126.1 | 495.7 ± 108.0 | 366.7 ± 122.3 | 507.2 ± 123.9 | 370.0 ± 155.1 | 448.4 ± 133.4 | 313.6 ± 124.3 |
| L3 | 460.6 ± 105.6 | 322.6 ± 140.7 | 488.4 ± 141.5 | 361.8 ± 122.6 | 495.7 ± 111.6 | 341.4 ± 133.6 | 467.1 ± 125.9 | 335.7 ± 134.2 |
| L4 | 508.0 ± 123.2 | 346.3 ± 146.2 | 475.3 ± 138.8 | 314.4 ± 113.6 | 489.2 ± 142.0 | 346.3 ± 129.0 | 497.4 ± 141.8 | 363.4 ± 155.3 |
| L5 | 480.2 ± 104.7 | 389.6 ± 128.7 | 473.7 ± 127.3 | 348.7 ± 122.9 | 486.7 ± 147.0 | 364.2 ± 133.4 | 496.5 ± 122.4 | 358.5 ± 130.2 |
| | | | | | | | | |
| 48h after | CTR | LC | CTR | LC | CTR | LC | CTR | LC |
| Th1 | 378.1 ± 135.1 | 321.0 ± 126.1 | 387.9 ± 116.5 | 334.0 ± 140.5 | 411.6 ± 128.7 | 339.7 ± 149.7 | 364.2 ± 119.4 | 319.3 ± 122.5 |
| Th3 | 369.1 ± 129.0 | 352.0 ± 135.0 | 423.9 ± 159.7 | 366.7 ± 140.9 | 411.6 ± 126.6 | 370.8 ± 170.5 | 365.1 ± 127.2 | 303.0 ± 118.6 |
| Th5 | 370.8 ± 133.3 | 347.1 ± 115.6 | 396.9 ± 146.6 | 378.9 ± 142.3 | 399.4 ± 159.1 | 387.1 ± 145.9 | 348.7 ± 114.6 | 338.1 ± 112.4 |
| Th7 | 393.6 ± 104.6 | 365.9 ± 157.5 | 399.4 ± 134.0 | 379.8 ± 127.3 | 398.5 ± 122.2 | 392.8 ± 145.3 | 409.2 ± 145.4 | 370.0 ± 163.9 |
| Th9 | 410.0 ± 113.9 | 384.7 ± 128.3 | 464.7 ± 151.2 | 426.3 ± 165.4 | 480.2 ± 158.0 | 421.4 ± 128.3 | 415.7 ± 140.6 | 418.1 ± 141.4 |
| Th11 | 437.7 ± 122.4 | 361.8 ± 98.2 | 490.0 ± 162.6 | 432.0 ± 110.4 | 471.2 ± 141.6 | 445.1 ± 157.3 | 404.3 ± 134.4 | 375.7 ± 146.9 |
| L1 | 460.6 ± 119.7 | 392.8 ± 180.1 | 537.4 ± 125.3 | 463.1 ± 157.9 | 557.8 ± 153.0 | 468.0 ± 203.8 | 453.3 ± 112.2 | 403.4 ± 143.4 |
| L2 | 455.7 ± 94.3 | 398.5 ± 188.1 | 542.3 ± 119.2 | 459.8 ± 177.9 | 529.2 ± 126.9 | 428.8 ± 141.6 | 440.2 ± 123.2 | 398.5 ± 161.4 |
| L3 | 463.9 ± 126.2 | 393.6 ± 160.3 | 481.8 ± 128.8 | 433.7 ± 155.1 | 511.2 ± 119.6 | 461.4 ± 163.6 | 498.2 ± 130.1 | 435.3 ± 188.8 |
| L4 | 512.1 ± 107.2 | 406.7 ± 201.3 | 497.4 ± 133.4 | 411.6 ± 126.2 | 519.4 ± 168.5 | 435.3 ± 136.4 | 532.5 ± 136.1 | 441.8 ± 164.1 |
| L5 | 494.9 ± 140.9 | 419.8 ± 136.6 | 504.7 ± 125.8 | 443.5 ± 117.2 | 512.1 ± 123.3 | 443.5 ± 153.7 | 530.0 ± 103.9 | 402.6 ± 152.8 |
PPTs in kPa are represented as mean ± SD (n = 12 participants each in the CTR and LC groups).
