# Supplementary material for: Pressure pain threshold map of thoracolumbar paraspinal muscles after lengthening contractions in young male asymptomatic volunteers
Source: Sci Rep. 2022 Sep 22;12:15825. doi: 10.1038/s41598-022-20071-4 (PMC9499944; doi:10.1038/s41598-022-20071-4)
Supplement: Supplementary file 3 — Supplementary Table 3. [file 41598_2022_20071_MOESM3_ESM.pptx]

## Slide 1
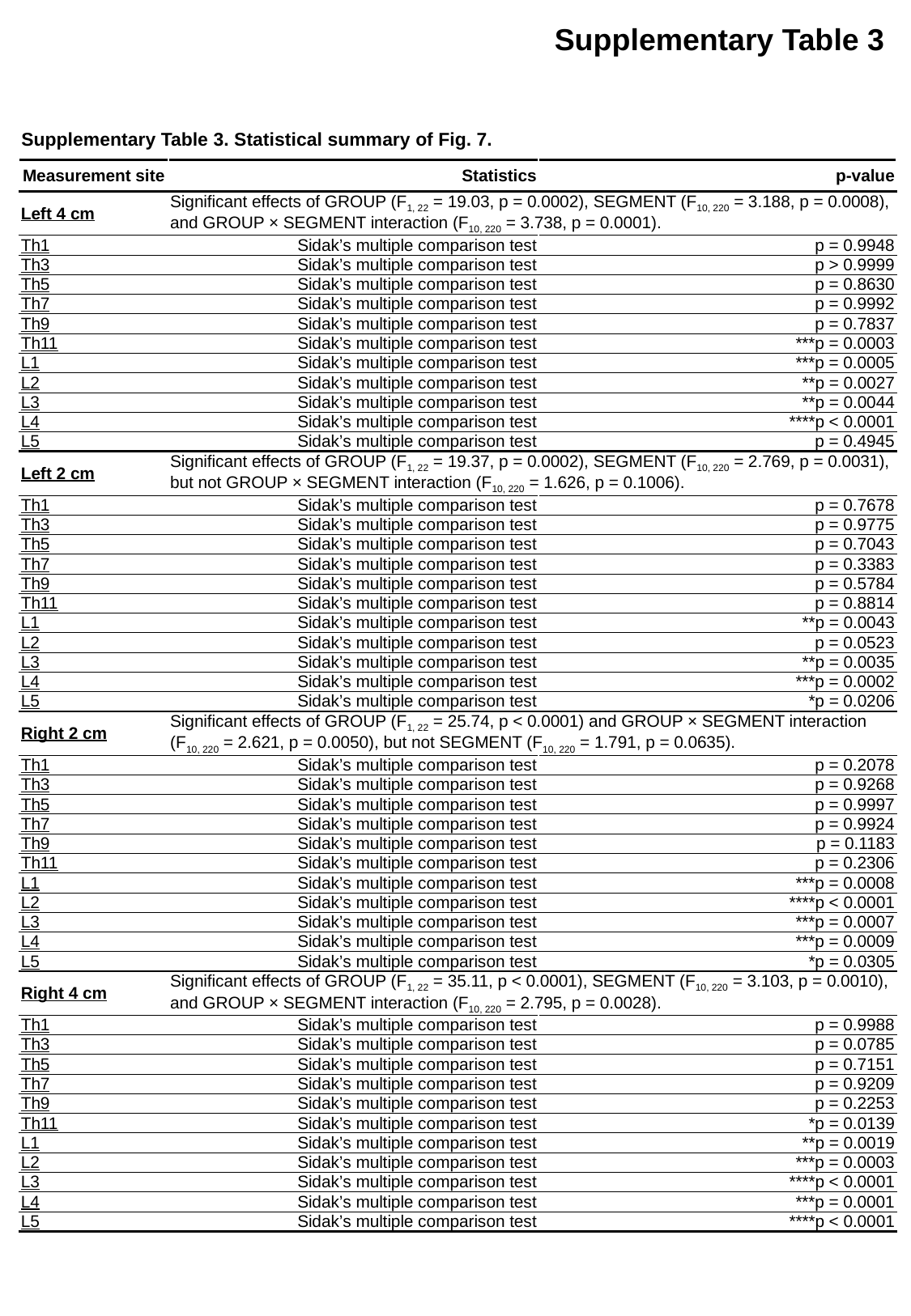

Supplementary Table 3
Supplementary Table 3. Statistical summary of Fig. 7.
| Measurement site | Statistics | p-value |
| --- | --- | --- |
| Left 4 cm | Significant effects of GROUP (F1, 22 = 19.03, p = 0.0002), SEGMENT (F10, 220 = 3.188, p = 0.0008), and GROUP × SEGMENT interaction (F10, 220 = 3.738, p = 0.0001). | |
| Th1 | Sidak’s multiple comparison test | p = 0.9948 |
| Th3 | Sidak’s multiple comparison test | p > 0.9999 |
| Th5 | Sidak’s multiple comparison test | p = 0.8630 |
| Th7 | Sidak’s multiple comparison test | p = 0.9992 |
| Th9 | Sidak’s multiple comparison test | p = 0.7837 |
| Th11 | Sidak’s multiple comparison test | \*\*\*p = 0.0003 |
| L1 | Sidak’s multiple comparison test | \*\*\*p = 0.0005 |
| L2 | Sidak’s multiple comparison test | \*\*p = 0.0027 |
| L3 | Sidak’s multiple comparison test | \*\*p = 0.0044 |
| L4 | Sidak’s multiple comparison test | \*\*\*\*p < 0.0001 |
| L5 | Sidak’s multiple comparison test | p = 0.4945 |
| Left 2 cm | Significant effects of GROUP (F1, 22 = 19.37, p = 0.0002), SEGMENT (F10, 220 = 2.769, p = 0.0031), but not GROUP × SEGMENT interaction (F10, 220 = 1.626, p = 0.1006). | |
| Th1 | Sidak’s multiple comparison test | p = 0.7678 |
| Th3 | Sidak’s multiple comparison test | p = 0.9775 |
| Th5 | Sidak’s multiple comparison test | p = 0.7043 |
| Th7 | Sidak’s multiple comparison test | p = 0.3383 |
| Th9 | Sidak’s multiple comparison test | p = 0.5784 |
| Th11 | Sidak’s multiple comparison test | p = 0.8814 |
| L1 | Sidak’s multiple comparison test | \*\*p = 0.0043 |
| L2 | Sidak’s multiple comparison test | p = 0.0523 |
| L3 | Sidak’s multiple comparison test | \*\*p = 0.0035 |
| L4 | Sidak’s multiple comparison test | \*\*\*p = 0.0002 |
| L5 | Sidak’s multiple comparison test | \*p = 0.0206 |
| Right 2 cm | Significant effects of GROUP (F1, 22 = 25.74, p < 0.0001) and GROUP × SEGMENT interaction (F10, 220 = 2.621, p = 0.0050), but not SEGMENT (F10, 220 = 1.791, p = 0.0635). | |
| Th1 | Sidak’s multiple comparison test | p = 0.2078 |
| Th3 | Sidak’s multiple comparison test | p = 0.9268 |
| Th5 | Sidak’s multiple comparison test | p = 0.9997 |
| Th7 | Sidak’s multiple comparison test | p = 0.9924 |
| Th9 | Sidak’s multiple comparison test | p = 0.1183 |
| Th11 | Sidak’s multiple comparison test | p = 0.2306 |
| L1 | Sidak’s multiple comparison test | \*\*\*p = 0.0008 |
| L2 | Sidak’s multiple comparison test | \*\*\*\*p < 0.0001 |
| L3 | Sidak’s multiple comparison test | \*\*\*p = 0.0007 |
| L4 | Sidak’s multiple comparison test | \*\*\*p = 0.0009 |
| L5 | Sidak’s multiple comparison test | \*p = 0.0305 |
| Right 4 cm | Significant effects of GROUP (F1, 22 = 35.11, p < 0.0001), SEGMENT (F10, 220 = 3.103, p = 0.0010), and GROUP × SEGMENT interaction (F10, 220 = 2.795, p = 0.0028). | |
| Th1 | Sidak’s multiple comparison test | p = 0.9988 |
| Th3 | Sidak’s multiple comparison test | p = 0.0785 |
| Th5 | Sidak’s multiple comparison test | p = 0.7151 |
| Th7 | Sidak’s multiple comparison test | p = 0.9209 |
| Th9 | Sidak’s multiple comparison test | p = 0.2253 |
| Th11 | Sidak’s multiple comparison test | \*p = 0.0139 |
| L1 | Sidak’s multiple comparison test | \*\*p = 0.0019 |
| L2 | Sidak’s multiple comparison test | \*\*\*p = 0.0003 |
| L3 | Sidak’s multiple comparison test | \*\*\*\*p < 0.0001 |
| L4 | Sidak’s multiple comparison test | \*\*\*p = 0.0001 |
| L5 | Sidak’s multiple comparison test | \*\*\*\*p < 0.0001 |
